# Time-Local Transformer

**DOI:** 10.1007/s42113-025-00253-9

**Published:** 2025-08-06

**Authors:** Billy Dickson, James Mochizuki-Freeman, Md Rysul Kabir, Zoran Tiganj

**Affiliations:** 1 Department of Computer Science, Indiana University Bloomington, Bloomington, Indiana USA; 2 Department of Psychological and Brain Sciences, Indiana University Bloomington, Bloomington, Indiana USA

**Keywords:** Transformer models, Working memory, Long-range dependencies, Neural plausibility, Language processing, Sequence modeling

## Abstract

Human language processing, characterized by its ability to capture long-range dependencies in sequential inputs, operates under the constraints of limited working memory. In contrast, state-of-the-art transformer models in artificial intelligence rely on access to the fixed context window, which deviates from the dynamic nature of human cognition. Here, we propose a novel approach to reconcile this disparity by integrating a computational model of working memory into the transformer architecture. This biologically-inspired modification constructs a time-local transformer, capable of learning complex dependencies without needing the full input history. Our findings demonstrate that this approach still preserves the capacity of transformers for effective sequence processing. This work is a step towards developing AI models that align more closely with the principles of human brain function, opening new avenues for understanding the neural underpinnings of language and cognition.

## Introduction

The human capacity for language acquisition and complex communication hinges on the brain’s ability to learn long-range dependencies in sequential data. In recent years, the field of artificial intelligence has seen remarkable advancements due to the development of transformer models, which excel in capturing complex long-range dependencies, enabling them to achieve excellent performance in NLP tasks. However, there exists a fundamental distinction in how transformers and the human brain approach language processing: transformers leverage their access to the fixed context window, enabling them to weigh the importance of different parts of the sequence at each processing step to capture statistical patterns. In contrast, human cognition operates under the constraints of limited working memory, dynamically processing incoming information without the luxury of storing and revisiting extensive past sequences. Here we demonstrate that a computational neuroscience model of working memory that gives rise to a timeline of the recent past can be used to substitute the need for a fixed context window. This makes it possible to build a time-local transformer, making this powerful architecture much more neurally plausible. Furthermore, the resulting timeline of the recent past is logarithmically compressed, meaning that the temporal resolution gradually decreases from the more recent towards the more distant past, consistent with Weber’s law (Weber, 1834). The logarithmic relationship implies that the amount of resources (in this case, neurons) grows logarithmically with the temporal extent of memory, making the representation rather scalable.

### Learning Long-Range Dependencies in Natural Language with Transformers

We start with an overview of the transformer architecture, introduced in Vaswani et al. (2017). Transformers have emerged as the dominant architecture in natural language processing (NLP) due to their exceptional ability to capture long-range dependencies in sequential data. At the core of a transformer lies the self-attention mechanism, which allows each element in the input sequence (e.g., a word in a sentence) to attend to all other elements in the sequence. This enables the model to weigh the importance of different parts of the input when generating a representation for each element, facilitating the capture of complex contextual relationships.

However, a key characteristic of the standard transformer architecture is its reliance on access to a long input sequence during processing, extending to tens of thousands of tokens in modern large language models (LLMs) like GPT-4 (with a context window exceeding 100,000 tokens, Achiam et al., 2023), Claude (with a context window exceeding 200,000 tokens, Anthropic, 2023), and Gemini (with capabilities to handle even sequences of 10 million tokens, Gemini Team et al., 2023). This allows the self-attention mechanism to consider all possible interactions between elements, but it deviates from the biological constraints of human cognition, where working memory limitations necessitate dynamic processing of incoming information without the ability to revisit extensive past sequences.

### Modeling Natural Language with RNNs

Prior to the advent of transformers, recurrent neural networks (RNNs) were the predominant architecture for modeling sequential data, including natural language. RNNs process input sequences one element at a time, maintaining a hidden state that summarizes the information from past elements. This hidden state is then combined with the current input to produce an output and update the hidden state for the next time step. The recurrent nature of RNNs allows them to capture temporal dependencies in the data, making them suitable for tasks like language modeling and machine translation.

However, standard RNNs can struggle to capture long-range dependencies due to the vanishing or exploding gradient problem during training (Bengio et al., 1994; Hochreiter, 1998). To address this limitation, attention mechanisms were introduced to RNNs (Bahdanau, 2014; Luong, 2015). In these models, an attention mechanism allows the RNN to selectively focus on relevant parts of the input sequence or its own hidden states when making predictions. This enables the model to access and weigh the importance of past information, even for elements far back in the sequence, thereby improving its ability to capture long-range dependencies.

While RNNs with attention offer improved capabilities for capturing long-range dependencies compared to standard RNNs, they remain neurally implausible. Even though RNNs process input sequentially, one word at a time, reminiscent of the temporal nature of human language processing, they rely on backpropagation through time (BPTT) for training. BPTT requires propagating error signals backward through the entire sequence, and is not considered neurally plausible as it requires a mechanism for storing and precisely reversing the sequence of computations. These limitations highlight the need for further exploration of alternative architectures and learning algorithms that align more closely with the principles of neural computation in the brain.

### Language Learning in Humans

The impressive human capacity for language learning unfolds from early childhood. Infants demonstrate an innate sensitivity to the statistical regularities of language, enabling them to segment continuous speech into words and extract grammatical patterns (Kuhl, 2004; Saffran et al., 1996). This early learning lays the foundation for subsequent vocabulary acquisition and syntactic development, facilitated by social interactions and exposure to rich linguistic environments (Senghas et al., 2004; Tomasello, 2005).

Crucially, the human brain does not store verbatim transcripts of past linguistic experiences. Instead, it dynamically processes incoming information, extracting meaning and constructing representations on-the-fly. This reliance on real-time processing, constrained by the limited capacity of working memory, suggests a departure from the mechanisms employed by transformer models, which have access to the input history. Understanding the neural computations underlying this dynamic language processing in humans remains an important challenge in cognitive neuroscience, with implications for both artificial intelligence and our understanding of the human mind.

### Transformers in Computational Neuroscience Models

The remarkable success of transformer architectures in artificial intelligence, particularly in capturing long-range dependencies, has spurred significant interest in exploring their potential relevance to neural computation (Parr et al., 2025). Several lines of research have attempted to bridge the gap between transformer mechanisms and biological plausibility. One prominent direction draws parallels between transformer operations and hippocampal function. It was demonstrated by Whittington et al. (2021), building on the Tolman-Eichenbaum Machine model (Whittington et al., 2020), that transformers equipped with recurrent position encodings can learn spatial representations akin to hippocampal place and grid cells. They highlight a mathematical relationship between the self-attention mechanism and memory retrieval processes in their hippocampal model, suggesting transformers might capture computational principles underlying spatial and relational memory. This perspective aligns with broader theories casting the hippocampus as implementing a form of key-value memory system, indexing content stored elsewhere. Gershman et al. (2025) elaborate on this, proposing a division of labor where the hippocampus handles keys (optimized for discriminability) and the neocortex handles values (optimized for fidelity).

Beyond hippocampal analogies, other work explores alternative biological substrates for transformer-like computations. Kozachkov et al. (2023) propose a novel mechanism involving neuron-astrocyte interactions, suggesting that tripartite synapses could implement the normalization step crucial to self-attention. They argue that astrocytes, modulating synaptic strength based on neural activity, provide a natural means to compute the complex, non-local operations inherent in transformers. Ellwood (2024) offers another perspective, showing how short-term, Hebbian-like synaptic plasticity could perform attention-like computations. In this “match-and-control” principle, spike train similarity (measured via calcium influx in spines) triggers transient, strong synaptic potentiation, allowing axons representing “keys” that match the somatic “query” to temporarily control the neuron’s output “value.” While these models differ in their proposed biological implementations–ranging from systems-level hippocampal circuits and key-value architectures to cellular mechanisms involving astrocytes or rapid plasticity–they collectively underscore the challenge and importance of reconciling transformer architectures with the time-local, memory-constrained nature of brain processing (Parr et al., 2025), a challenge our work directly addresses by integrating a specific computational model of working memory.

These findings suggest that transformers, despite their architectural differences from biological neural networks, might capture some essential computational principles underlying not only language processing but also spatial and relational memory. However, the reliance of standard transformers on the full input history remains a significant obstacle to their direct application in computational neuroscience models. The development of time-local transformers, which operate under constraints similar to those of human working memory, could provide a more neurally plausible framework for understanding the mechanisms of various cognitive functions, including language processing, spatial navigation, and memory formation.

### Intuition Behind Time-Local Transformer

To construct a more neurally plausible version of a transformer, we use the computational neuroscience memory model introduced in Shankar and Howard (2012). It extends a well-known temporal context model (TCM) of memory (Howard & Kahana, 2002), and it has been successful in describing a wide range of phenomena in neuroscience (Howard et al., 2014) including the emergence of time and place cells (Eichenbaum, 2014; Howard & Eichenbaum, 2015; Tiganj et al., 2017) as well as in cognitive psychology memory experiments such as free recall and judgment of recency (Howard et al., 2015; Maini et al., 2022; Palombo et al., 2019; Tiganj et al., 2021, 2022).

Electrophysiological recordings suggest that time cells are also sensitive to stimulus identity, encoding not just when something happened but also what happened (Cruzado et al., 2020; Omer et al., 2023; Tiganj et al., 2018). While these recordings were conducted on primates and bats, they indicate a potential capability of time cells to encode a mental timeline of log-compressed linguistic input. Importantly, time cells in these studies typically exhibited firing properties consistent with log-compression (Cao et al., 2022).

The model we propose here receives one token embedding at a time and feeds each individual embedding dimension into a set of RNN units with an analytically derived diagonal connectivity matrix. The fact that the connectivity matrix is diagonal implies that each RNN unit will act as a collection of leaky integrators with impulse responses that decay exponentially with a time constant determined by the recurrent weights. The weights are chosen such that the neurons decay at a spectrum of time constants, implementing a real-domain approximation of the Laplace transform. This is an integral transform that converts a function of time into a function of Laplace variable *s*. To construct an internal timeline of the recent past, we invert the Laplace transform, resulting in a set of neurons with sequentially activated bell-shaped impulse responses. This representation encodes a logarithmically compressed past because the width of the responses scales with the peak time. The timeline produced by the Laplace and inverse Laplace transform serves as an input to multi-head self-attention. We train attention weights using backpropagation with a cross-entropy loss computed at every time step between the network output and the next input token. In the following section, we provide implementation details for a time-local transformer based on the log-compressed memory timeline and then present the evaluation results on a dataset commonly used for training LLMs.

**Fig. 1:**
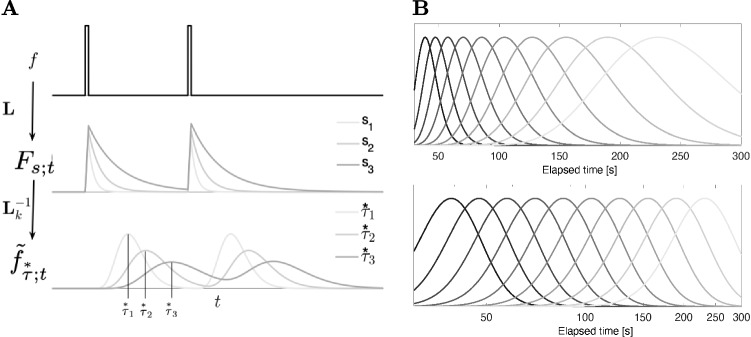
**A** Response of the neurons in memory timeline model to $\delta$ pulses. Neurons in $F_{s;t}$ decay exponentially at a spectrum of time constants *s* implementing a discrete approximation of a real-domain Laplace transform. Neurons in $\tilde{f}_{\overset{*}{\tau };s}$ activate sequentially resembling time cells. **B** A set of log-compressed impulse responses from ten different neurons resembling time cells. Log-compression is manifested in log-spaced peak times and constant coefficient of variation (the width of the response increases with the peak time). With the x-axis plotted as a function of the logarithm of time (bottom panel), the responses are uniformly spaced and have equal widths

## Methods

### Memory Timeline

Building on models from computational and cognitive neuroscience (Howard et al., 2014; Shankar & Howard, 2012), we designed a neural network architecture which stores the input sequence in the form of a log-compressed memory timeline. Specifically, this network constructs an approximation of a real-domain Laplace transform of the temporal history of the input signal and then constructs an approximate inverse of the history, giving rise to an internal timeline of the past.

To construct this memory representation, we use a network composed of two layers. The input coming from the encoder, which we label as *f*, is fed into a recurrent layer (*F*) with the weights analytically computed to approximate the real-domain Laplace transform of the temporal history of the input. The output of the recurrent layer is mapped through a linear layer with analytically computed weights implementing the inverse Laplace transform $\tilde{f}$. We describe this procedure step-by-step below, first in continuous-time and then in its discrete, neural network implementation.

#### Continuous-Time Formulation of the Laplace and Inverse Laplace Transform

Given a one-dimensional input signal *f*(*t*), we define a modified version of the Laplace transform *F*(*s*; *t*):

1 $$\begin{aligned} F(s;t) = \int _{0}^t e^{-s\left( t - t' \right) }f(t') dt'. \end{aligned}$$

This modified version differs from the standard Laplace transform only in the variable *s*. Instead of *s* being a complex value composed of real and imaginary parts, we restrict *s* to a positive real value. This modification simplifies the neural network implementation while giving us the computational benefits of the standard Laplace transform, as illustrated below. Note that *F* is also a function of time *t*. This implies that at every moment, we construct the Laplace transform of the input function up to time *t*: $f(0 \le t'<t) \xrightarrow {L} F(s;t)$.

To construct the temporal history of the input, we need to invert the Laplace transform. The inverse which we denote as $\tilde{f}(\overset{*}{\tau };t)$ can be computed using Post’s inversion formula (Post, 1930):

2 $$\begin{aligned} \tilde{f}(\overset{*}{\tau };t) = \textbf{L}^{-1}_kF(s;t) = \frac{(-1)^k}{k!} s^{k+1} \frac{d^k}{ds^k}F(s;t), \end{aligned}$$

where $\overset{*}{\tau }:= k/s$ and $k \rightarrow \infty$. As shown below, we will compute a discrete approximation of the inverse and use a finite value of *k*.

#### Neural Networks Implementation of the Laplace and Inverse Laplace Transform

To describe a neural network approximation of the Laplace transform, we first rewrite Eq. 1 in a differential form:

3 $$\begin{aligned} \frac{dF(s;t)}{dt} = -s F(s;t) + f(t). \end{aligned}$$

The impulse response (response to input $f(t) = \delta (0)$) of *F*(*s*; *t*) decays exponentially as a function of time *t* with decay rate *s*: $e^{-st}$ (Fig. 1A). Note that this is a linear transform, so *F*(*s*; *t*) will be a convolution between *f*(*t*) and the impulse response.

We implement an approximation of the modified Laplace and inverse Laplace transform as a two-layer neural network with analytically computed weights. The first layer implements the modified Laplace transform through an RNN. The second layer implements the inverse Laplace transform as a dense layer with weights analytically computed to implement a *k*-th order derivative with respect to *s*.

While in the Laplace domain *s* is a continuous variable, here we redefine *s* as a vector of *N* elements. We can now write a discrete-time approximation of Eq. 3 as an RNN with a diagonal connectivity matrix and a linear activation function:

4 $$\begin{aligned} F_{s;t} = \textbf{L} F_{s;t-1} + f_t , \end{aligned}$$

where $\textbf{L}:= e^{-{\text {diag}}(s)\Delta t}$ is an $N \times N$ matrix implementing the discrete Laplace transform operator. At every time step *t*, $F_{s;t}$ is an *N*-element vector. For brevity of notation, we assume that the duration of a discrete-time step $\Delta t = 1$. Importantly, since the values of vector ${\textbf {s}}$ are fixed, $\textbf{L}$ is also fixed and not learned during training.

Following Eq. 2, a discrete approximation of the inverse Laplace transform, $\tilde{f}_{\overset{*}{\tau };t}$, can be implemented as a dense layer on top of $F_{s;t}$. Rather than using Post inversion formula and *k*-th order numerical differentiation as in Shankar and Howard (2012), we found that better numerical stability can be obtained using a Gaver-Stehfest method for numerical approximation of the inverse transform (Gaver Jr, 1966; Stehfest, 1970) as implemented in Horváth et al. (2020) (see also Tano et al. (2020); Abate and Whitt (2006) for additional approaches to computing the inverse transform).

To interpret $\tilde{f}_{\overset{*}{\tau };t}$ and to select *s* values in an informed way, we compute the impulse response of $\tilde{f}_{\overset{*}{\tau };t}$. For input $f(t) = \delta (0)$, the activity of $\tilde{f}_{\overset{*}{\tau };t}$ is:

5 $$\begin{aligned} \tilde{f}_{\overset{*}{\tau };t} = \frac{1}{t} \frac{k^{k+1}}{k!} \left( \frac{t}{\overset{*}{\tau }}\right) ^{k+1} e^{-k\frac{t}{\overset{*}{\tau }}}. \end{aligned}$$

The impulse responses of units in $\tilde{f}_{\overset{*}{\tau };t}$ are a set of unimodal basis functions (Fig. 1). To better characterize its properties, we first find the peak time by taking a partial derivative with respect to *t*, equate it with 0 and solve for *t*: $\partial \tilde{f}_{\overset{*}{\tau };t}/ \partial t = 0 \rightarrow t=\overset{*}{\tau }$. Therefore each unit in $\tilde{f}_{\overset{*}{\tau };t}$ peaks at $\overset{*}{\tau }$. Note that if we computed the exact continuous-time Laplace and inverse Laplace transform (which would require infinitely many neurons since *s* and $\mathbf {\overset{*}{\tau }}$ would be continuous variables), the impulse response would be a $\delta (\overset{*}{\tau })$. This would provide a perfect reconstruction of the input function $f(0<t'<t)$ rather than its approximation.

To further characterize our approximation, we express the width of the unimodal basis functions of the impulse response of $\tilde{f}_{\overset{*}{\tau };t}$ through the coefficient of variation *c* (see Appendix for the derivation of *c*): $c=1/\sqrt{k+1}$. Importantly, *c* does not depend on *t* and $\overset{*}{\tau }$, implying that the width of the unimodal basis functions increases linearly with their peak time. Therefore, when observed as a function of $\log (t)$, the width of the unimodal basis functions is constant.

We choose values of $\mathbf {\overset{*}{\tau }}$ as log-spaced between some minimum and maximum. Because of the log-spacing and because *c* does not depend on *t* and $\overset{*}{\tau }$, when analyzed as a function of $\log (t)$, the unimodal basis functions are equidistant and equally wide, providing uniform support over the $\log (t)$ axis (Fig 1B). This result is analogous to the scale-invariance observed in human timing and perception, formalized as Weber’s law. Intuitively, this is beneficial since the more recent past carries more predictive power than the more distant past. Hence, our approximation of the function $f(0<t'<t)$ will be better for values closer to *t* than to 0. Note that fixing the values of $\overset{*}{\tau }$ and choosing *k* also fixes values of *s* since $s=k/\overset{*}{\tau }$, so *s* is not a trainable parameter.

**Fig. 2:**
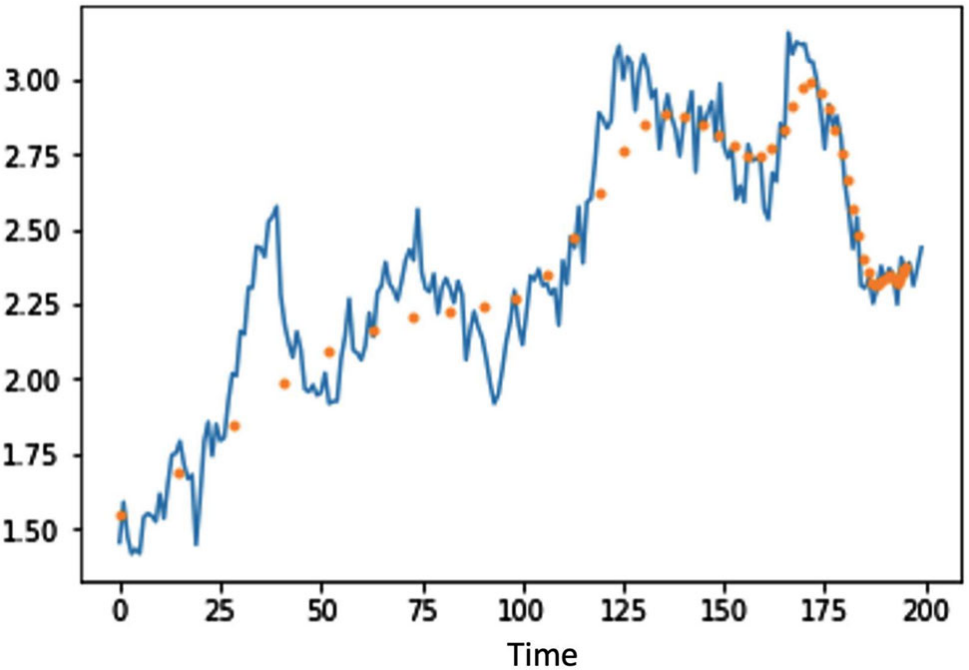
Time-local memory representation stores log-compressed timeline of the input signal. Activation of $\tilde{f}$ neurons at time 200 as a function of their peak time (orange dots) for a time-series input (blue line). Since $\tilde{f}$ neurons have sequentially activated log-compressed impulse responses, their output is a log-compressed memory representation of the input signal such that activation of $\tilde{f}$ neurons at time 200 approximates values of the input function at different times in the past with highest temporal resolution for the more recent past

Since the Laplace and inverse Laplace transforms are linear, the output of the system is equivalent to the convolution of the impulse responses of $\tilde{f}$ with the input signal. Because the impulse responses constitute a log-compressed sequence of bell-shaped temporal basis functions, their activity will constitute a log-compressed memory of the input signal between minimum and maximum values of $\overset{*}{\tau }$. This is illustrated in Fig. 2 where a time-varying input is fed into the network and activity of $\tilde{f}$ at the end of the input sequence is plotted as a function of the peak time. The activity of $\tilde{f}$ stores the log-compressed version of the signal, with resolution gradually decaying from more recent towards more distant past.

**Fig. 3:**
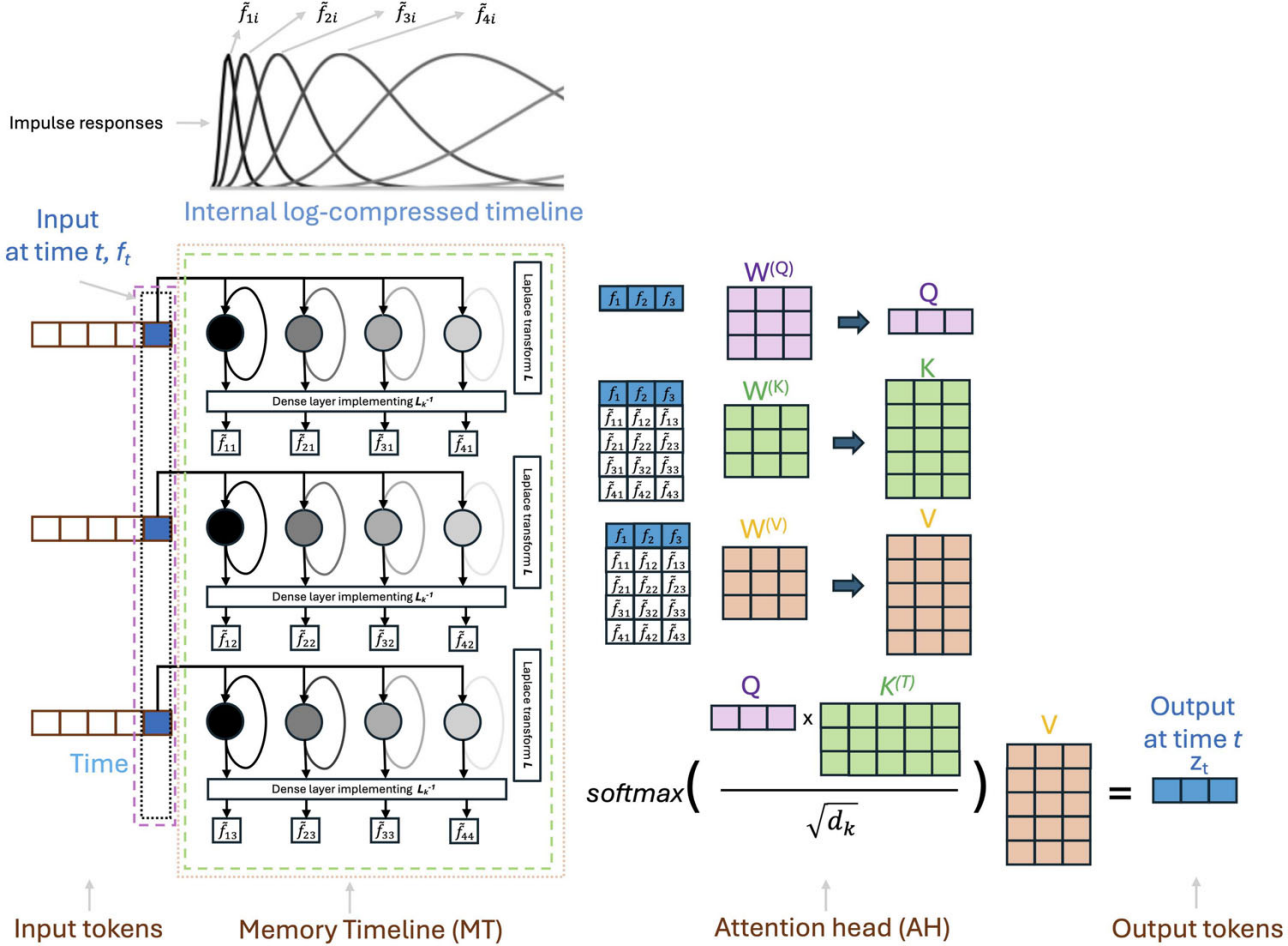
Schematic of one head of a time-local transformer. Time series $\textbf{f}_t$ of input token embedding is fed into the log-compressed temporal memory. The schematic shows three dimensions of the embedding, each being stored into a separate temporal memory module. Each module is depicted with $N = 4$ capacity consisting of recurrently connected neurons that implement the real-domain Laplace transform $\textbf{L}$ and that project into the dense layer implementing the inverse transform $\textbf{L}^{-1}_k$. Neurons $\tilde{f}$ have scale-invariant sequentially activated impulse responses (depicted on the top). Outputs of the temporal memory are used by the attention head to produce query, key, and value vectors. The output of the attention layer is computed using Eq. (6) as illustrated in the figure. Note that the output vector $\textbf{z}_t$ is updated at every time step and that its size matches the size of the input vector $\textbf{f}_t$ enabling stacking of multiple layers

### Time-Local Transformer Architecture

We now describe all the components of the time-local transformer architecture, including the memory timeline with token inputs, attention heads, and integration of these components into a multi-head multi-layer architecture.

#### Encoding the Input Tokens into Memory Timeline

We now consider a sequence of tokenized words where each token is embedded in *D* dimensions. We split the input embedding into $d=D/n$ groups, where *n* is the number of transformer heads. Each token dimension is fed as input *f*(*t*) into a separate memory timeline module. As described above, each memory timeline module produces a set of $\tilde{f}_{i,j}$ values that encode a log-compressed memory of the input sequence between $\overset{*}{\tau }_{min}$ and $\overset{*}{\tau }_{max}$ time in the past where *i* goes from 1 to *N* (the number of $\overset{*}{\tau }$ values) and *j* goes from 1 to *d*.

#### Applying Attention on Memory Timeline

In standard transformer architecture, the entire input sequence is used to generate query, key, and value matrices that are then combined into attention scores. To keep our architecture time-local, we use only the currently processed token and the content of the memory timeline to compute attention scores. Specifically, query vector *Q* (size *d* by 1) is generated as a product between the *d*-dimensional part of the input embedding and the query weights matrix $W^{(Q)}$. Key and value matrices *K* and *V* (both of size $N+1$ by *d*) are generated as products between a matrix constructed by concatenating *d*-dimensional part of input embedding with the corresponding part of the memory timeline $\tilde{f}$ and weights matrices $W^{(K)}$ and $W^{(V)}$ (Fig. 3). Note that a standard transformer would have a query matrix instead of a query vector, with the same dimension as key and value matrices. Computing the queries as vectors at every time step enables time-local operations while preserving the basic computational properties of transformers. Unlike standard transformers that attend to the entire input sequence, our time-local transformer attends to a compressed representation of the past, maintained in the memory timeline, thus operating without direct access to the entire context window.

**Fig. 4:**
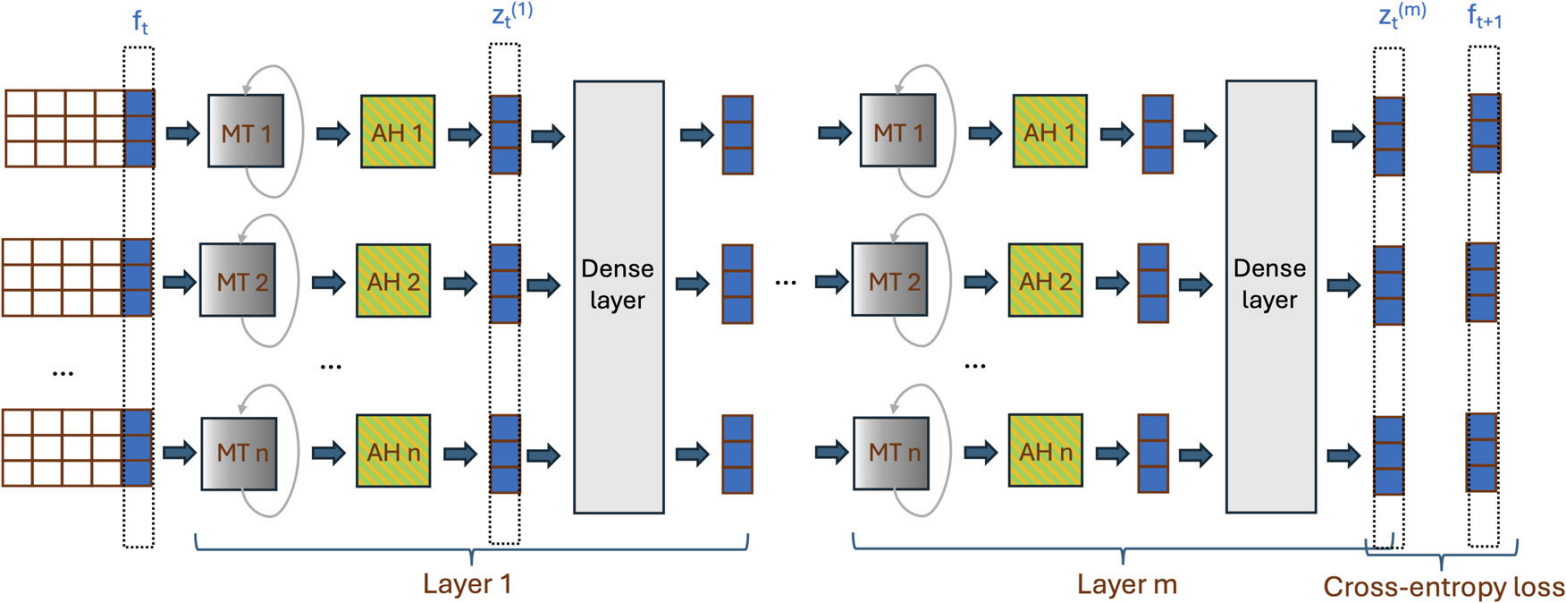
Schematic of the multi-layer and multi-head time-local transformer architecture. The figure illustrates architecture with *m* layers each consisting of *n* heads. Note that input token embedding is split into *n* equal size parts, each of which is processed by a separate attention head. The outputs from these heads are then combined and projected back to the original input size via a dense layer. The training objective is a cross-entropy loss calculated between the final layer’s normalized output and the embedding of the actual next token $\textbf{f}_{t+1}$

We compute the output of an attention head by following the commonly used approach that combines keys, queries, and values:

6 $$\begin{aligned} \text {attention output} = \text {softmax}\left( \frac{QK^T}{\sqrt{d_k}}\right) V \end{aligned}$$

In the above equation, the product between *Q* and the transpose of *K* is scaled down by the square root of the dimensionality of the keys, $\sqrt{d_k}$, in our case $d_k = N+1$. The scaled scores are subsequently passed through a softmax function to normalize them into a probability distribution. Finally, these normalized attention scores are used to perform a weighted summation of the values *V*.

#### Constructing Multi-head Multi-layer Time-Local Transformer

The overall architecture follows principles common to modern, high-performance transformer models (often referred to as Transformer++ architectures), with specific design choices mirroring the Llama 3.1 architecture (Grattafiori et al., 2024). The attention computation described above is independently replicated across each of the *n* attention heads. The outputs from all heads are combined via projection through a weight matrix $W^{(Z)}$. Within each transformer block, the input embedding to the multi-head attention sublayer and the subsequent input to the feed-forward network (FFN) sublayer undergoes Root Mean Square Layer Normalization (RMSNorm) (Zhang & Sennrich, 2019). Residual connections are employed around both sublayers.

The FFN sublayer in each transformer block uses the SwiGLU (Swish Gated Linear Unit) activation function (Shazeer, 2020). The size of the intermediate hidden dimension of the FFN is determined by first scaling the model’s embedding dimension ($d_{\text {model}}$) by a factor of $\frac{8}{3}$, applying an additional multiplier, 1.3, and then rounding the result up to the nearest multiple of 256.

The SwiGLU operation computes:

7 $$\begin{aligned} \text {SwiGLU}(x, W, V, W_2) = (\text {SiLU}(xW) \odot xV) W_2 \end{aligned}$$

where *x* is the input (which has already passed through RMSNorm in this context), *W* and *V* are weight matrices projecting to the calculated intermediate dimension, $W_2$ projects back to $d_{\text {model}}$, $\text {SiLU}(z) = z \cdot \sigma (z)$ is the Sigmoid Linear Unit activation (with $\sigma$ being the sigmoid function), and $\odot$ denotes element-wise multiplication.

After processing through all transformer layers, the final output undergoes one last RMSNorm application. This normalized output is then projected by the final language modeling head to produce logits that are subsequently passed through a softmax function to generate the probability distribution over the vocabulary for predicting the next token (Fig. 4). The predicted probability of the next token is used to compute the loss via a cross-entropy function. The loss is then backpropagated through the model to update the parameters. To ensure time-locality by preventing backpropagation through time, the previous hidden state is detached from the computation graph after each token. By doing this, we prevent gradients from flowing through time, ensuring that the model’s updates are based solely on the current and past states as represented in the memory timeline. We accumulate the gradients and update the weights after every 32,768 tokens.

### Comparative Analysis with Standard RNN, Laplace Forward Pass, and Traditional Transformer Self-Attention

To evaluate the utility of employing fixed-weight recurrent units for generating a memory timeline, we performed three comparative experiments. First, we substituted our specialized *F* and $\tilde{f}$ units with those of a standard simple RNN architecture, featuring randomly initialized trainable weights instead of fixed ones. Second, we tested a configuration using only the forward Laplace transform component (the *F* nodes) and omitting the inverse transform component (the $\tilde{f}$ nodes). Lastly, we tested traditional transformer fixed-window self-attention, which we refer to as the “Shift Register” model. In this model, given some number of nodes, *N*, the current token attended to itself and the *N* immediately preceding tokens, mimicking standard causal self-attention. The surrounding model architecture was held constant across all comparisons.

### Training

We trained our models on the Wikitext-2 dataset (Merity et al., 2016), a standard benchmark for language model evaluation derived from Wikipedia articles. This dataset contains approximately 2.45M training tokens, 259k validation tokens, and 296k test tokens. For tokenization, we utilized the EleutherAI GPT-Neo tokenizer (Gao et al., 2020) with its vocabulary size adjusted to 50,304 (originally 50,257) for efficiency. Our model configurations shared core parameters: an embedding dimension of 192, 6 attention heads, and an FFN size of 768. We varied the model depth, experimenting with 1, 2, and 4 layers. Optimization was performed using Adam (Kingma & Ba, 2017) ($\beta _1=0.9$, $\beta _2=0.999$). The learning rate was set at $10^{-3}$ with decay to $10^{-4}$ over 18 epochs. For implementing the Gaver-Stehfest method, we set the maximum function evaluations parameter to 8. When implementing the Laplace-only model, we normalized F using the inverse hyperbolic sine for improved numerical stability. To ensure robustness, each model configuration was trained three times with different random weight initializations and data shuffling. All models converged over the course of training, reaching a point where perplexity on the validation set began to increase.

## Results

We evaluated a time-local transformer based on a memory timeline network using the computational neuroscience model from Shankar and Howard (2012). We also assessed three control networks: a time-local transformer based on a simple RNN, one using only the Laplace transform without its inverse, and one using a shift register, similar to a traditional transformer self-attention where at each timestep the current token attends to each of the past *N* number of tokens. The models ranged in size from approximately 19.9M to 21.9M parameters.

To characterize the contribution of different parts of the memory timeline, we conducted the evaluation on different numbers of $\tilde{f}$ units, *N*. For each linear step increase in *N*, we doubled $\overset{*}{\tau }_{max}$. For example, with $N=2$, $\overset{*}{\tau }_{max}$ was 2 and peak times of $\tilde{f}$ filters were [1, 2], with $N=3$, $\overset{*}{\tau }_{max}$ was 4 and peak times of $\tilde{f}$ filters were [1, 2, 4], and following this pattern, with $N=5$, $\overset{*}{\tau }_{max}$ was 16 with $\tilde{f}$ filter peak times of [1, 2, 4, 8, 16].

As an evaluation metric, we use perplexity, which is a quantitative measure of how well a probability model predicts a sample. In the context of language models, it reflects the ability of the model to anticipate the next word in a sequence, with lower perplexity scores indicating better predictive performance. Perplexity is defined as the exponentiated average negative log-likelihood of the sequence:

$$\begin{aligned} Perplexity = e^{-\frac{1}{N_{t}} \sum _{i=1}^{N_{t}} \log P(w_i)} \end{aligned}$$

where $N_{t}$ is the number of tokens in the sequence and $P(w_i)$ denotes the probability assigned by the model to the *i*-th token. When comparing two models on the same dataset, a lower perplexity score indicates that the model assigns a higher overall likelihood to the test sequence, suggesting a better statistical fit to the data. In our experiments, we ensure the reliability of the observed differences by averaging perplexity scores over three independent training runs with varying random initializations, confirming that observed trends are consistent and not due to random variation.

**Fig. 5:**
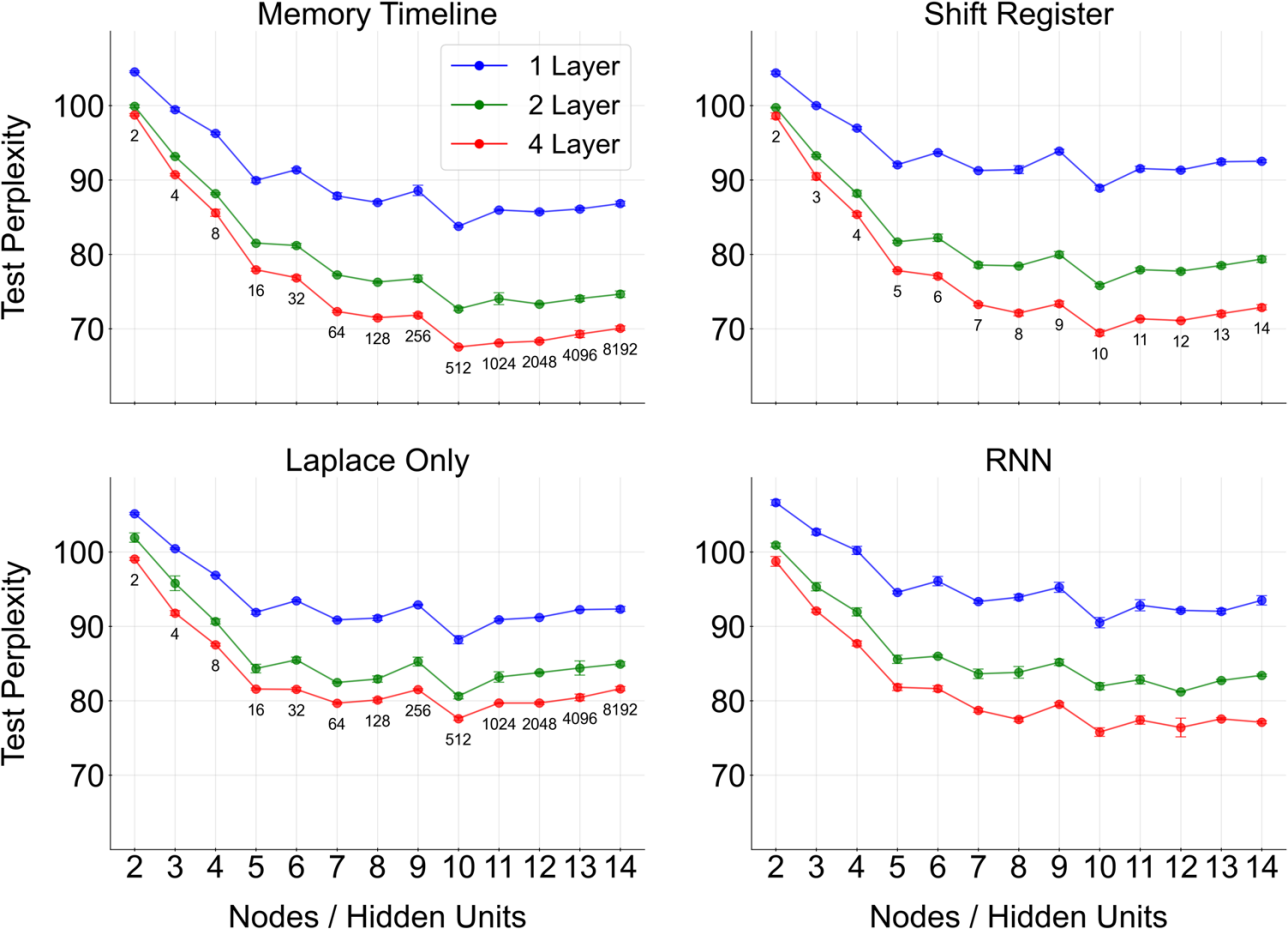
Test perplexity on Wikitext-2 for different model architectures and layers, as a function of the number of nodes/units (N). For Memory Timeline and Laplace Only, N is the number of $\tilde{f}$ or *F* units, respectively, corresponding to exponentially increasing $\tau _{\max }$ (values indicated for Memory Timeline). For the Shift Register, N is the size of the attention look-back window. For RNN, N is the number of hidden units. Lower perplexity indicates better performance

**Fig. 6:**
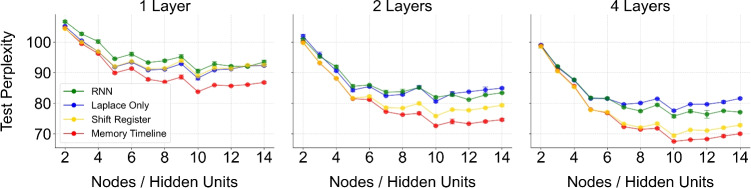
Comparison of test perplexity across model types for 1, 2, and 4 layers on Wikitext-2 (same results as in Fig. 5 but with a direct comparison of the four different models)

Figure 5 shows the perplexity for the memory timeline architecture as well as the three comparison architectures. Figure 6 compares model performance by depth.

For each version of the model, perplexity gradually decreased for the first $N=10$ of $\tilde{f}$ units and then plateaued or increased. $N=10$ corresponds to $\overset{*}{\tau }_{max}=512$, indicating that including around 512 tokens into the memory timeline improves the prediction. Perplexity plateauing for $\overset{*}{\tau }_{max} > 512$ suggests the model struggles to utilize information from more distant tokens to predict the next token. This could be due to the diminishing predictive power of distant words in Wikitext-2 or the broader smoothing window caused by log-compression in the memory timeline. Increasing the number of layers consistently improved performance across all configurations, and a similar pattern was observed across each depth.

In our control analysis, replacing the Laplace and inverse Laplace transform that gives rise to the memory timeline with a simple RNN resulted in a similar decreasing and then plateauing pattern. The RNN does not store an explicit memory timeline (different nodes in the RNN do not naturally capture specific temporal windows) and performed worse than the memory timeline model. Additionally, we experimented with using only the *F* units instead of $\tilde{f}$, resulting in similar performance to the RNN, likely because the inverse transform is essential for constructing a temporally organized representation. The shift register notably overlapped in performance with the memory timeline model up to 6 hidden units, indicating that adding additional hidden units in the memory timeline improves performance over adding additional units in the shift register.

## Discussion

We addressed the problem of making transformer architecture more neurally plausible. While transformers have demonstrated exceptional performance in various domains, their reliance on access to the entire context window during attention deviates from the biological constraints of the human brain. We address this gap by incorporating a computational model of working memory timeline into the transformer architecture, enabling it to operate with a dynamically updated context.

Learning using prediction error has been remarkably influential in neuroscience and machine learning (Friston, 2010). In the brain, prediction errors, or the discrepancies between expected and actual sensory inputs are thought to drive learning and adaptation (Rao & Ballard, 1999). Similarly, in machine learning, many powerful algorithms including backpropagation rely on minimizing prediction errors to update model parameters. By designing a transformer that operates with a dynamically updated context window, analogous to the constraints of working memory, we created a model that learns by continuously updating its predictions based on incoming information. This approach enhances the biological plausibility of the transformer architecture. Moreover, the logarithmic compression of the timeline, a feature inherent to our working memory model, aligns with Weber’s law (Weber, 1834; Portugal & Svaiter, 2011), potentially leading to improved generalization over longer time scales.

Our results indicate that increasing the buffer size beyond 512 tokens does not lead to improvements in model performance. We attribute this to the artificial structure of datasets typically employed for training large language models. For instance, the Wikitext dataset used here consists of randomly ordered Wikipedia articles, inherently limiting correlations to within-article contexts and preventing effective utilization of long-range dependencies across article boundaries. This contrasts markedly with natural human experience, which exhibits extensive long-range correlations and dependencies across scales spanning hours, days, weeks, or even months, as evidenced by recent empirical studies using ecological data collection methods (Altmann et al., 2012; Tanaka-Ishii et al., 2018; Kello et al., 2010; Sreekumar et al., 2014; Nielson et al., 2015; Yim et al., 2023). These studies consistently show that human experience–whether in language, memory retrieval, or daily behavior–features significant correlations across extended timescales, thereby suggesting that more ecologically valid data might better leverage our model’s capacity for long-range temporal correlations.

Our primary objective was to propose a time-local implementation of transformers, increasing the biological plausibility of such architecture. The log-compression that emerges from the proposed approach and gives rise to a scale-free representations is supported by a number of studies, including electrophysiology and neuroimaging work, that strongly support the presence of long-range temporal correlations and scale-free (1/f) neural dynamics, aligning with the properties of our model. For instance, seminal research has revealed logarithmic time representations in rodent hippocampal time cells (Cao et al., 2022), scale-free neural dynamics in human cortical electrophysiological recordings (Miller et al., 2009), and hyperbolic integration of past events in retrosplenial cortex activity (Danskin et al., 2023). Additional studies have established that diverse cortical areas, including retrosplenial cortex, prefrontal cortex, and hippocampus, exhibit power-law distributed temporal dynamics consistent with long-range dependencies (Beggs & Plenz, 2003; Linkenkaer-Hansen et al., 2001; Palva et al., 2013; Poil et al., 2012; Petermann et al., 2009; He, 2010; Tagliazucchi et al., 2012). Collectively, these empirical findings support the neural plausibility of employing logarithmic compression and long-range temporal correlations within our computational architecture.

The problem of time-locality in the context of biological learning systems is also present in self-supervised contrastive learning (Chen et al., 2020), specifically in contrastive learning through time (CLTT) (Schneider et al., 2021). CLTT uses a principle of temporal slowness (Wiskott & Sejnowski, 2002) aiming to bring embeddings of temporally close image frames closer in the embedding space, while distancing frames that are further apart in time. Such approach can help with initial learning or pretraining of neural networks and improve subsequent downstream performance on image classification problems. Access to a memory timeline could enable learning systems like CLTT to distinguish between inputs that occurred close in time from those that occurred further apart, all within a neurally plausible framework.

The memory timeline has also been utilized in reinforcement learning to construct an estimate of the future as a function of future time (Howard et al., 2023; Masset et al., 2023; Momennejad & Howard, 2018; Tano et al., 2020; Tiganj et al., 2019). By learning the average memory timeline for different stimuli, one can predict the average timeline of future events for any of those stimuli, enabling time-local learning of temporal relationships and evaluation of expected future reward. A similar framework has been used in reinforcement learning to build systems that can learn to represent variables such as numerosity and position (Maini et al., 2023; Mochizuki-Freeman et al., 2024). This is done by modulating the rate of exponential decay of units in *F* by some learned variables from the environment. This effectively modulated the speed of the sequential activation in $\tilde{f}$, turning it into sequential activation over the temporal derivative of the learned variable rather than time.

The utility of the log-compressed timeline in machine learning has been demonstrated with systems that, while not time-local, utilized the impulse responses of $\tilde{f}$ to perform temporal convolution (Jacques et al., 2021, 2022). These systems demonstrated strong capability in learning long-range temporal dependencies on time-series prediction benchmarks. A similar approach with temporal convolution could potentially be applied to the language modeling task explored in this work. While our primary objective was to enhance the neural plausibility of transformers, the desirable properties of log-compression could be leveraged in a performance-optimized language model. Such a model could sacrifice time-locality for increased computational efficiency by replacing the RNN implementation of the Laplace transform with temporal convolution using $\tilde{f}$ impulse responses. Additionally, the recent success of state-space models in NLP (Dao & Gu, 2024; Fu et al., 2022; Gu et al., 2022, 2021; Gu & Dao, 2023; Mehta et al., 2023; Lenz et al., 2025) and other time-local frameworks (Voelker et al., 2019; Chilkuri et al., 2021) offers further avenues for building time-local learning systems for language processing.

## Conclusions

We have demonstrated that a computational neuroscience model of working memory based on a log-compressed timeline can serve as a neural basis for a time-local transformer architecture. Our objective was not to construct an architecture that is competitive in terms of performance on machine learning benchmarks, but to demonstrate how using a computational neuroscience model can convert a widespread machine learning architecture into one that is closer to biological plausibility. Our results indicate that the Laplace framework can give rise to a memory timeline that can replace the attended sequence of a standard transformer. Furthermore, the logarithmic compression inherent in this timeline offers efficient scaling, as the neural resources required grow only logarithmically with sequence length, making this approach viable for representing extended temporal contexts.

## Data Availability

No datasets were generated or analysed during the current study.

## Appendix. Computing Coefficient of Variation of $\tilde{f}$

$\tilde{f}$ has a unimodal impulse response with peak at $t=\overset{*}{\tau }$. The coefficient of variation, *c*, is a ratio of standard deviation and mean. The mean of $\tilde{f}$ is:

$$\begin{aligned} \mu&= \int _0^\infty t \tilde{f}(s;t) dt \\&= \int _0^\infty t \frac{1}{t} \frac{k^{k+1}}{k!} \left( \frac{t}{\overset{*}{\tau }}\right) ^{k+1} e^{-k\frac{t}{\overset{*}{\tau }}} dt \\&= \frac{k^{k+1}}{k!} \int _0^\infty \left( \frac{t}{\overset{*}{\tau }}\right) ^{k+1} e^{-k\frac{t}{\overset{*}{\tau }}} dt \\&= \overset{*}{\tau }\frac{k+1}{k}. \end{aligned}$$

The standard deviation of $\tilde{f}$ is:

$$\begin{aligned} \sigma&= \sqrt{\int _0^\infty (t-\mu )^2 \tilde{f}(s;t) dt} \\&= \sqrt{\int _0^\infty (t-\mu )^2 \frac{1}{t} \frac{k^{k+1}}{k!} \left( \frac{t}{\overset{*}{\tau }}\right) ^{k+1} e^{-k\frac{t}{\overset{*}{\tau }}} dt}\\&= \overset{*}{\tau }\frac{\sqrt{k+1}}{k}. \end{aligned}$$

Finally, the coefficient of variation is then:

$$\begin{aligned} c = \frac{\sigma }{\mu } = \frac{1}{\sqrt{k+1}}. \end{aligned}$$

The coefficient of variation depends only on *k*. Therefore, the variance grows with the peak time. In other words, variance is constant as a function of the logarithm of the peak time.

## Code Availability

The code is available at https://github.com/cogneuroai/time-local-transformer.
